# Circadian clock regulation in lung health and disease: molecular mechanisms and therapeutic opportunities

**DOI:** 10.17179/excli2026-9372

**Published:** 2026-04-08

**Authors:** Prabesh Baniya, Swekriti Puri, Nisha Panth, Rajan Thapa, Madhu Gupta, Sachin Kumar Singh, Rohit Bhatia, Gaurav Gupta, Kamal Dua, Keshav Raj Paudel

**Affiliations:** 1 Department of Biomedical Science and Engineering, Gwangju Institute of Science and Technology, Gwangju 61005, Republic of Korea; 2 JF Institute of Health Sciences, Tribhuvan University, Kathmandu 44600, Nepal; 3 Discipline of Pharmacy, Graduate School of Health, University of Technology Sydney, Ultimo, New South Wales, Australia; 4 Department of Pharmacy, Sungkyunkwan University, Suwon 16419, Republic of Korea; 5 School of Pharmaceutical Sciences, Delhi Pharmaceutical Science and Research University (DPSRU), Pushp Vihar, New Delhi 110017, India; 6 School of Pharmaceutical Sciences, Lovely Professional University, Phagwara 144411, India; 7 Chitkara College of Pharmacy, Chitkara University, Rajpura, Punjab, India; 8 Centre for Research Impact & Outcome-Chitkara College of Pharmacy, Chitkara University, Rajpura, 140401, India; 9 Centre of Medical and Bio-Allied Health Sciences Research, Ajman University, Ajman, United Arab Emirates; 10 NICM Health Research Institute and School of Science, Western Sydney University NSW 2145, Australia

**Keywords:** circadian rhythm, clock gene, lung disease, chronotherapy

## Abstract

Circadian rhythms are endogenous time-keeping mechanisms that organize physiological and cellular functions into approximately 24-hour cycles. These rhythms are generated by conserved molecular clocks composed of interconnected transcription-translation feedback loops involving core regulators such as CLOCK, BMAL1, PERIOD, CRYPTOCHROME, REV-ERB, and ROR proteins. While circadian regulation is best known for governing sleep-wake behavior, mounting evidence demonstrates that peripheral clocks exert critical control over organ-specific physiology. The lung represents a highly rhythmic tissue in which local circadian oscillators coordinate airway tone, immune surveillance, inflammatory signaling, epithelial repair, and metabolic homeostasis. Disruption of circadian timing arising from genetic alterations, environmental stressors, shift work, irregular light exposure, or chronic inflammation has emerged as an important contributor to the development and progression of multiple respiratory diseases, including asthma, chronic obstructive pulmonary disease, pulmonary fibrosis, acute lung injury, sleep-disordered breathing, and lung cancer. At the mechanistic level, dysregulation of clock genes alters inflammatory pathways, oxidative stress responses, mitochondrial metabolism, and cell-cycle control, thereby exacerbating tissue injury and impairing resolution and repair. Recognition of these temporal influences has prompted growing interest in circadian-based therapeutic strategies. Chronotherapy, which aligns drug administration with endogenous biological rhythms, and pharmacological targeting of clock components such as REV-ERBs and RORs, offer promising avenues to improve treatment efficacy while limiting adverse effects. This review integrates current insights into circadian clock architecture, lung-specific regulation, disease mechanisms, and therapeutic potential, highlighting circadian biology as a critical yet underutilized dimension of respiratory medicine.

See also the graphical abstract(Fig. 1).

## Introduction

Circadian rhythms are internally driven cycles that last roughly 24 hours and are fundamental in coordinating different physiological functions, such as the sleep-wake pattern, metabolic activity, hormonal regulation, and immune system dynamics (Foster, 2020[43]). The phenomenon of leaf movements in the Mimosa plant, which continued to open and close rhythmically even in constant darkness, was first documented in 1729 by the French astronomer Jean Jacques d'Ortous de Mairan. This early observation pointed to the existence of an internal biological clock. Centuries later, definitive genetic evidence came from the pioneering work of Ron Konopka and Seymour Benzer, who identified three mutations in a single functional gene on the X chromosome of Drosophila melanogaster that disrupt its normal 24-hour behavioral rhythms (Konopka and Benzer, 1971[82]; Van Laake et al., 2018[157]).

Jeffrey Hall and Michael Rosbash later provided the molecular characterization of the period (PER) gene, demonstrating that it produces the PER protein, whose RNA and protein levels exhibit rhythmic fluctuations across the circadian cycle (Zehring et al., 1984[176]). Subsequent investigations uncovered several other genes that regulate circadian rhythms, leading to the recognition that a restricted set of “core clock genes” serves as the central machinery of biological timekeeping. These genes generate rhythmic patterns of transcription and translation that sustain endogenous oscillations. A pivotal advance was made by Hardin, Hall and Rosbash (1990) (Hardin et al., 1990[60]), who showed that the *per* gene in Drosophila regulates its own mRNA expression through a feedback mechanism in which the PER protein ultimately represses its transcription. This finding provided the first molecular evidence for how circadian oscillations are maintained. The suprachiasmatic nucleus (SCN) in the hypothalamus acts as the central pacemaker, coordinating daily rhythms across the body by synchronizing peripheral clocks in other tissues (Welsh et al., 2010[168]). Environmental light cues are conveyed to the brain's master circadian clock by intrinsically photosensitive retinal ganglion cells, which relay photic signals to the suprachiasmatic nucleus through the retinohypothalamic pathway (Fernandez et al., 2016[42]).

In mammals, circadian rhythms are generated by a transcription-translation feedback loop (TTFL), in which the transcription factors Circadian Locomotor Output Cycles Kaput (CLOCK) and Brain and Muscle ARNT-Like 1 (BMAL1) act as core activators, inducing the expression of downstream clock genes including *Per1, Per2, Per3*, and the cryptochromes (*Cry1* and *Cry2*), whose protein products subsequently repress their own transcription to complete the feedback cycle (Shearman et al., 1997[146]; Kume et al., 1999[86]). The CLOCK-BMAL1 complex activates nuclear receptors Reverse Strand of ERBA (REV-ERB) and Retinoic Acid Receptor-Related Orphan Receptor (ROR), which competitively bind Retinoic Acid Receptor-Related Orphan Receptor Response Element (RORE) in Bmal1 and Clock promoters to regulate their transcription in a secondary feedback loop. REV-ERBs repress, while RORs activate gene expression, generating transcriptional oscillations essential for circadian rhythm (Guillaumond et al., 2005[56]). Post-translational modifications, including phosphorylation and acetylation, modulate the stability, functional activity, and interactions of CLOCK proteins, while coordinated protein-protein interactions orchestrate multiprotein complex formation to precisely regulate circadian transcription and sustain robust rhythmicity in the mammalian clock (Narasimamurthy and Virshup, 2021[119]).

Temporal regulation* via *circadian rhythms is pivotal in optimizing diverse physiological functions, including metabolism, immune responses, cellular repair processes, neurobehavioral activities, and notably, the pathophysiology of pulmonary diseases (Neves et al., 2022[120]). Disruption of circadian rhythms, attributable to lifestyle factors such as shift work, irregular sleep-wake cycles, exposure to inconsistent light-dark patterns, or genetic abnormalities affecting clock gene expression, is implicated in the onset and progression of numerous pathological states (Lecacheur et al., 2024[90]). Substantial epidemiological and mechanistic studies have linked circadian misalignment to increased susceptibility to metabolic disorders, including type 2 diabetes and obesity, cardiovascular diseases, psychiatric conditions such as depression, various cancers, and neurodegenerative illnesses like Alzheimer's and Parkinson's disease (Neves et al., 2022[120]).

At the molecular level, circadian disruption disturbs the oscillatory expression of clock-controlled genes and regulatory hormones, which in turn perturbs fundamental biological processes such as the cell cycle, DNA repair mechanisms, and inflammatory responses. This dysregulation exacerbates disease pathogenesis by fostering environments conducive to oxidative stress, chronic inflammation, and aberrant cellular proliferation (Wilking et al., 2013[169]). Circadian misalignment in humans raises C-Reactive Protein (CRP), interleukin-6, resistin, and Tumor Necrosis Factor alpha (TNFα), which drive hypertension through inflammation and endothelial dysfunction (Douma and Gumz, 2018[32]). Importantly, circadian regulation operates in an organ-specific manner, highlighting its multifaceted influence on physiological integrity. Cardiovascular function demonstrates pronounced diurnal variation influenced by circadian modulation of autonomic nervous system activity and vascular tone (Helissen et al., 2023[61]). Consistent with this concept, Rev-erbα emerges as a circadian regulator of neuronal excitability, where its upregulation enhances seizure susceptibility via modulation of GABAergic signaling and disrupts diurnal seizure rhythmicity, highlighting how clock-controlled pathways influence oxidative stress, synaptic balance, and epilepsy vulnerability in the central nervous system (Zhang et al., 2021[178]; Huang et al., 2024[65]). On the other hand, experimental disruption of the core circadian clock gene period markedly accelerates neurodegeneration, shortens lifespan, worsens motor decline, and enhances oxidative damage in aging Drosophila, indicating a direct neuroprotective role of circadian rhythms independent of disease-specific pathways (Krishnan et al., 2012[85]).

The focus on lung diseases and therapeutic perspectives in relation to clock genes and circadian rhythms is driven by an increasing recognition of how intrinsic biological clocks profoundly influence lung function and disease pathophysiology. Circadian rhythms, regulated by molecular clock genes such as *Bmal1, Pers, Crys, and Rev-erbs*, orchestrate a wide array of physiological processes within the respiratory system, including cellular immune responses, inflammation, tissue repair, and airway resistance. Disruption of these rhythms adversely affects lung biology and contributes significantly to the development and progression of chronic pulmonary diseases such as asthma, chronic obstructive pulmonary disease (COPD), pulmonary fibrosis, cystic fibrosis, pulmonary arterial hypertension, and lung cancer (Giri et al., 2022[53]).

Lung function itself exhibits robust circadian variation, with symptoms and physiological parameters such as airway resistance, inflammatory cell numbers, and respiratory tolerance fluctuating over a 24-hour cycle. Night-time and early morning rises in airway resistance have long been recognized as a feature of chronic inflammatory lung conditions, including asthma and COPD (Agusti et al., 2011[1]; Scheer et al., 2021[144]). This temporal variation in lung physiology highlights the critical role of clock gene regulation in maintaining respiratory homeostasis and suggests that circadian dysregulation exacerbates lung disease symptomatology and severity. Clock gene disruption is mechanistically linked to increased oxidative stress, inflammation, dysregulated immune-inflammatory responses, mucus secretion, and abnormal tissue remodeling in lung diseases (Sundar et al., 2015[151]). Experimental evidence demonstrates that loss of the core circadian regulator Bmal1, or disturbance of circadian rhythms through jet lag, was found to worsen acute viral bronchiolitis triggered by Sendai virus (SeV) and influenza A virus in mice (Ehlers et al., 2018[36]). Disruption of circadian timing has been demonstrated to weaken pulmonary defense mechanisms by compromising epithelial barrier properties and reducing vascular stability, thereby increasing the lung's vulnerability to injury and infection. (Casanova et al., 2024[18]). Such findings underscore how the circadian clock functions as a critical regulator of pulmonary immune defense and tissue homeostasis.

From a therapeutic perspective, targeting circadian clock pathways represents a promising frontier for developing novel treatments. Pharmacological modulation of clock genes and their downstream molecular pathways may restore rhythmicity and alleviate the pathological traits observed in chronic lung diseases. Newly developed compounds that specifically modulate circadian proteins like REV-ERBs and RORs show promise in restoring rhythm balance and enhancing respiratory function (Giri et al., 2022[52]). The emerging discipline of circadian medicine capitalizes on these insights, advocating chrono-therapeutic approaches in which the administration of pharmacological agents is timed to coincide with the body's endogenous rhythms (Dallmann et al., 2014[26]). Such strategies aim to maximize therapeutic efficacy while minimizing adverse effects. Personalized medicine that considers an individual's chronotype and circadian profile promises to refine disease management and prevention, fostering more effective and tailored interventions.

## The Molecular Architecture of Circadian Clocks

Circadian clocks are internal biological timing mechanisms that regulate daily rhythms in physiological and behavioral functions. These rhythms are produced by conserved molecular systems that control time-dependent gene expression within cells. In mammals, circadian regulation is organized as a coordinated network, where a central pacemaker aligns the activity of peripheral clocks present in different tissues (Fagiani et al., 2022[40]). This integrated molecular framework enables precise temporal control of biological processes through the rhythmic expression of clock-controlled genes.

### Core transcriptional-translational feedback loops

The molecular machinery of the circadian clock is primarily driven by TTFL, which functions as a self-sustaining oscillator to maintain near-24-hour rhythmicity in gene and protein expression. These loops represent one of the most conserved mechanisms across evolution, underlying the temporal organization of behavior and physiology in mammals. At the core of this system are positive and negative regulatory arms that generate rhythmic oscillations by alternating cycles of transcriptional activation, protein accumulation, inhibition, and degradation (Shearman et al., 1997[146]).

The positive limb of the central loop is composed of the transcription factors CLOCK and BMAL1. These proteins form heterodimers that bind to E-box DNA sequences in the promoter regions of target genes, particularly those encoding the *Per1, Per2, Per3,* and *Cry1, Cry2* families (Gekakis et al., 1998[50]). Once transcribed, *Per* and *Cry* mRNAs are exported to the cytoplasm and translated into proteins. Initially, PER and CRY proteins accumulate gradually, but as they reach sufficient levels, they assemble into complexes that are actively transported back into the nucleus. Within the nucleus, these complexes act as repressors by directly inhibiting the transcriptional activity of CLOCK-BMAL1, thereby suppressing further production of their own transcripts (Lee et al., 2001[91]; Duong et al., 2011[34]). This delayed negative feedback ensures that transcriptional activity oscillates over approximately 24 hours.

The cycle is reset when PER and CRY proteins undergo phosphorylation by kinases such as casein kinase 1δ/ε, targeting them for ubiquitination and proteasomal degradation. Their removal relieves the repression of CLOCK-BMAL1, initiating a new round of transcription (Lee et al., 2001[91]). This sequence of activation, repression, and degradation constitutes the primary circadian feedback loop and is responsible for the rhythmic rise and fall of core clock gene expression. Importantly, the inherent delay between transcriptional activation and the nuclear repressive action of PER-CRY complexes is what allows the system to approximate a full day.

Beyond the core feedback loop, additional regulatory mechanisms contribute to the stability and accuracy of circadian rhythms. Key players in this process include REV-ERBα, REV-ERBβ, and the ROR family members RORα, RORβ, and RORγ. Both groups of regulators interact with ROREs in the Bmal1 promoter, but with opposite outcomes: RORs promote BMAL1 transcription, while REV-ERBs inhibit it. This dynamic balance allows precise control of BMAL1 expression and further stabilizes the circadian oscillations (Sato et al., 2004[142]; Guillaumond et al., 2005[56]). Furthermore, multiple core clock genes contain ROREs within their promoter regions, highlighting the stabilizing influence exerted by the opposing actions of RORs and REV-ERBs (Ueda et al., 2005[155]). On a broader scale, transcriptomic and gene expression studies across different tissues and cell types consistently point to a central role for these nuclear receptors in shaping circadian gene regulation (Lau et al., 2008[88]; Jetten, 2009[71]).

**Figure 2:**
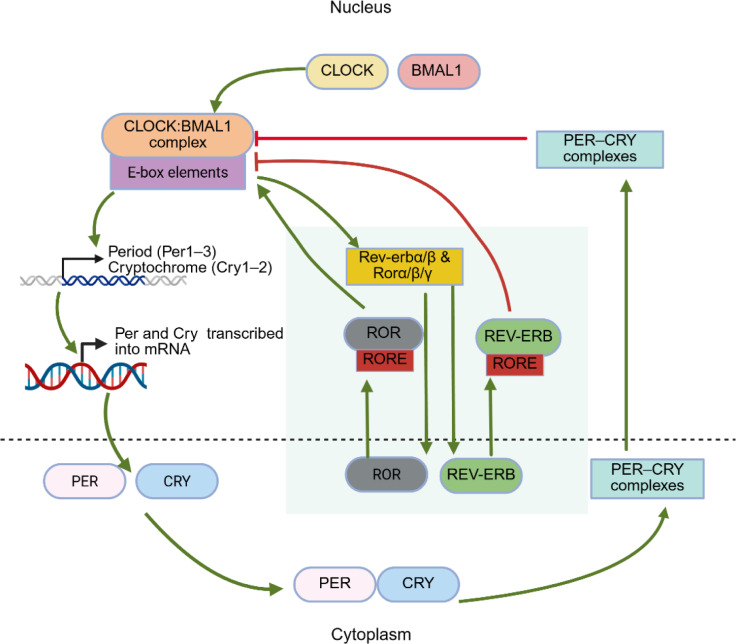
The mammalian circadian rhythm is governed by a transcription-translation feedback loop (TTFL). Within the nucleus, CLOCK and BMAL1 proteins form a heterodimer that binds to E-box sequences in DNA, initiating the transcription of Period (Per1-3) and Cryptochrome (Cry1-2) genes. PER and CRY proteins then accumulate in the cytoplasm, assemble into complexes, and return to the nucleus, where they inhibit the CLOCK: BMAL1 complex, thereby suppressing their own transcription. A secondary loop involves the same CLOCK: BMAL1 complex promoting the expression of Rev-erbα/β and Rorα/β/γ, which compete for binding to RORE elements in the Bmal1 promoter. ROR proteins enhance Bmal1 transcription, while REV-ERBs suppress it, adding another layer of control that reinforces the stability of the clock. Collectively, these linked feedback mechanisms maintain rhythmic gene expression over an approximately 24-hour cycle. Image was drawn using BioRender.com

These TTFL are not confined to the master circadian pacemaker in the SCN of the hypothalamus. They are also active in peripheral tissues, including the liver, lungs, heart, and kidneys, where they synchronize local physiology to systemic timing cues (Hunter and Bechtold, 2025[67]). In this way, TTFL orchestrate temporal regulation of a large proportion of the genome, influencing diverse processes such as sleep-wake cycles, hormone secretion, metabolism, immune responses, and even cellular repair mechanisms (Fagiani et al., 2022[40]). Disruptions in these regulators have been associated with various health issues, such as sleep disorders, metabolic dysfunction, cardiovascular problems, and cancer, highlighting their critical role in maintaining overall health (Merikanto et al., 2013[112]; Knutson and von Schantz, 2018[80]; Baldanzi et al., 2022[7]; Her et al., 2024[62]). The core transcription-translation feedback loop of the circadian clock is shown in Figure 2(Fig. 2).

## Peripheral Clocks in the Lung and Interaction with the Central Clock

The circadian system consists of two main components: the central clock in the brain's SCN and peripheral clocks present in nearly all tissues and organs. Light detected by the retina travels via the retinohypothalamic tract as electrical signals, which the SCN then translates into chemical messages. These light cues, together with physiological factors such as meal timing, work to synchronize the central circadian clock (Albrecht, 2004[2]). Two primary theories describe how the central circadian clock interacts with peripheral clocks. The first, known as the “master-slave” model, proposes that the SCN central clock exerts complete control over synchronization, with peripheral clocks entirely dependent on the central clock and unresponsive to other external or internal signals. The second, referred to as the hierarchical network model, portrays the central clock as a conductor and peripheral clocks as individual contributors, highlighting coordinated yet partly independent regulation by each peripheral clock. This model allows peripheral clocks, such as those in the kidney, liver, and lungs, to respond dynamically to both internal and external cues while still being influenced by light signals transmitted from the central clock (Dibner et al., 2010[28]). The lung, serving as a crucial interface between the external environment and internal physiology, exhibits strong circadian rhythms essential for respiratory function, immune defense, and overall homeostasis. Within the lung, a network of cell-intrinsic circadian oscillators operates through the canonical transcription-translation feedback loop, including CLOCK, BMAL1, PER1-3, CRY1-2, REV-ERBα/β, and ROR proteins, producing ~24-hour cycles of gene expression in airway epithelial cells, alveolar type II cells, vascular endothelium, fibroblasts, and resident immune cells (Gibbs et al., 2014[51]; Han et al., 2021[59]; Wu et al., 2022[170]). These clocks orchestrate diurnal variation in pulmonary processes such as immune cell recruitment, cytokine release, airway responsiveness, surfactant secretion, and xenobiotic metabolism. Recent research indicates that various environmental exposures, such as air pollutants and particulates, xenobiotic metabolism, cigarette smoke, disrupted sleep schedules from shift work or jet lag, altered oxygen levels, ventilator-associated injury, and microbial infections, can interfere with the lung's molecular clock and accelerate the onset of respiratory disorders (Gebel et al., 2006[49]; Davidson et al., 2009[27]; Logan et al., 2012[102]; Li et al., 2014[94]; Pekovic-Vaughan et al., 2014[127]; Pilorz et al., 2014[129]). 

Although the SCN serves as the master pacemaker, lung oscillators are synchronized indirectly through rhythmic systemic cues, including glucocorticoids, autonomic inputs, body-temperature cycles, and feeding-related signals (Pezuk et al., 2012[128]; Sundar et al., 2015[151]). Lesions of the SCN or exposure to constant light are known to dampen or shift circadian oscillations, such as Per2::Luc rhythms, in peripheral tissues, including the lung. Conversely, systemic cues like scheduled glucocorticoid administration or restricted feeding can re-entrain pulmonary clocks even when the SCN remains intact, highlighting the capacity of strong peripheral inputs to override central timing signals. While the predominant flow of circadian information is from the SCN to the lung, there is emerging evidence that systemic factors feed back to the central pacemaker. For instance, pro-inflammatory cytokines such as interleukin-6 and tumor necrosis factor-α released during immune activation can indirectly influence SCN activity and transiently adjust behavioral rhythms. (Pezuk et al., 2012[128]; Izumo et al., 2014[70]; Husse et al., 2015[68]; Shlykova et al., 2023[147]).

### Epigenetic regulation of circadian clocks function in the lungs 

While transcription-translation feedback loops drive the molecular circadian oscillator, additional regulatory layers are required to generate tissue-specific rhythmic gene expression in peripheral organs such as the lung. Among these regulatory layers, epigenetic mechanisms play a fundamental role in modulating the accessibility of circadian gene promoters and coordinating the temporal organization of transcriptional programs (Papazyan et al., 2016[124]). Chromatin remodeling processes, including histone acetylation, histone methylation, and DNA methylation, allow circadian transcription factors to regulate gene expression in a rhythmic and context-dependent manner (Raduwan et al., 2013[134]; Yuan et al., 2024[174]; Nie and Menet, 2025[121]). 

Circadian gene promoters exhibit time-dependent histone acetylation, particularly at H3K9 and H3K14, within regions that control clock-regulated genes (Doi et al., 2006[31]). These rhythmic acetylation patterns are associated with the activity of the histone acetyltransferase **p300** and with the intrinsic acetyltransferase function of the **CLOCK** protein itself, linking chromatin modification directly to circadian transcriptional regulation (Etchegaray et al., 2003[38]; Doi et al., 2006[31]). These modifications facilitate the recruitment of transcriptional machinery and allow rhythmic activation of genes that regulate diverse physiological functions. Opposing enzymatic activities further refine these transcriptional oscillations. Histone deacetylases, particularly SIRT1, regulate circadian transcription by removing acetyl groups from histones and core clock proteins, leading to reduced transcriptional activity during specific phases of the circadian cycle (Nakahata et al., 2008[117]). Since SIRT1 activity depends on intracellular NAD⁺ availability, this mechanism provides a direct link between circadian gene regulation and the cell's metabolic state (Nakahata et al., 2009[118]). In pulmonary tissues, such metabolic-epigenetic coupling is particularly important, as the lung is constantly exposed to fluctuating environmental conditions, including oxygen availability, pollutants, and inflammatory stimuli (Gibbs et al., 2014[51]). Through coordinated cycles of acetylation and deacetylation, chromatin states alternate between transcriptionally active and repressive configurations, ensuring that circadian genes are expressed with precise timing.

Beyond histone acetylation dynamics, histone methylation also contributes to circadian regulation in lung cells (González-Suárez and Aguilar-Arnal, 2024[55]). Methyltransferases and demethylases modify specific lysine residues on histone tails, thereby influencing nucleosome stability and transcription factor accessibility (González-Suárez and Aguilar-Arnal, 2024[55]). The histone methyltransferase MLL1 promotes trimethylation of histone H3 at lysine 4 (H3K4me3) at promoters of clock-controlled genes, a chromatin mark generally linked to active transcription (Katada and Sassone-Corsi, 2010[77]). This modification supports the binding and transcriptional activity of the CLOCK-BMAL1 complex, thereby increasing circadian gene expression during the transcriptionally active phase. In contrast, the Polycomb-associated enzyme EZH2 generates the repressive histone mark H3K27me3, which promotes chromatin condensation and reduced transcription at certain clock gene regions (Aranda et al., 2015[6]). The histone demethylase KDM5A contributes to the dynamic regulation of methylation at these promoters, enabling daily fluctuations in histone modification states (Dimitrova et al., 2015[29]). Together, the coordinated activities of MLL1, EZH2, KDM5A, and related chromatin-modifying enzymes allow clock gene promoters to alternate between transcriptionally permissive and repressive chromatin configurations over the 24-hour cycle, helping maintain stable circadian rhythms in gene expression.

DNA methylation within CpG-rich regions is another mechanism that can influence circadian gene regulation. For example, increased methylation at the promoter of BMAL1 has been shown to reduce its transcriptional activity (Satou et al., 2013[143]). Patterns of DNA methylation in circadian genes are not fixed and may change under conditions such as aging, metabolic disorders, or disturbances of normal sleep cycles. These epigenetic alterations can affect circadian system function, leading to reduced rhythm amplitude, shifts in circadian phase, and weakened or irregular oscillations in rhythmic gene expression.

Circadian regulation in the lung is closely linked to the control of immune responses and inflammatory signaling. Many immune mediators and cytokines exhibit time-of-day-dependent expression patterns, reflecting the influence of circadian clock components on pulmonary immune cell activity (Sundar et al., 2015[151]). Disruption of circadian rhythms, caused by factors such as shift work, irregular sleep patterns, or chronic inflammation, can impair these regulatory mechanisms and lead to dysregulated immune responses in the respiratory system (Fagiani et al., 2022[40]). Consequently, circadian disruption has been associated with increased susceptibility to respiratory diseases, including Asthma and Chronic Obstructive Pulmonary Disease (Giri et al., 2022[53]). These conditions often display pronounced diurnal variation in symptom severity, with many patients experiencing worsening airway obstruction or inflammation during specific times of the day or night.

**Figure 3:**
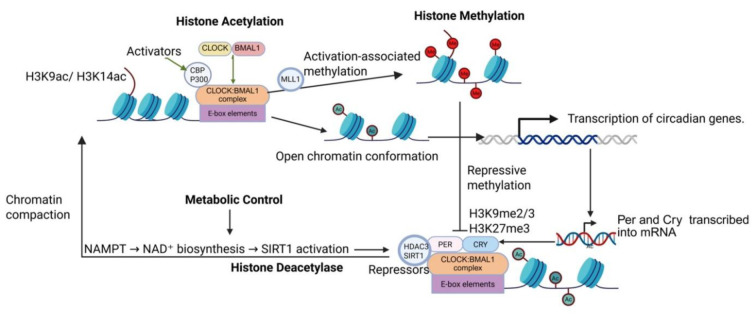
The transcription factors CLOCK and BMAL1 form a heterodimer that binds to E-box elements within the promoters of circadian genes. This complex recruits co-activators such as CBP/p300 and the histone methyltransferase MLL1, leading to histone acetylation (Ac) and methylation (Me) that promote an open chromatin structure and active transcription. As PER and CRY proteins accumulate, they associate with the CLOCK: BMAL1 complex and recruit repressors including HDAC3 and SIRT1, resulting in histone deacetylation and transcriptional repression. The coordinated balance between these activating and repressive chromatin modifications generates rhythmic circadian gene expression. Image was drawn using BioRender.com.

Understanding the epigenetic mechanisms that regulate circadian clock function in lung tissues has significant clinical implications. Insights into how chromatin modifications influence circadian transcription may reveal new therapeutic opportunities aimed at restoring rhythmic physiological processes in the respiratory system. Emerging strategies such as chronotherapy, which involves administering treatments according to circadian timing, have gained increasing attention as potential approaches for improving treatment efficacy while minimizing adverse effects. As research continues to uncover the molecular links between circadian clocks, epigenetic regulation, and pulmonary physiology, targeting circadian pathways may represent a promising avenue for the development of innovative therapies for respiratory diseases. Epigenetic control of circadian gene expression is shown in Figure 3(Fig. 3). 

### Clock-controlled genes and their relevance to lung physiology

The lung circadian clock orchestrates daily physiological rhythms, particularly regulating inflammation, and its disruption, triggered by factors such as pollutants, infections, smoking, shift work, or stress, has been linked to abnormal immune responses contributing to diseases like asthma, COPD, and pulmonary fibrosis (Sundar and Srinivasan, 2021[150], Li et al., 2022[95]; Wang et al., 2023[167]). Inflammatory regulation is tightly controlled by core clock genes such as BMAL1, which activate PER (PER1-3) and CRY (CRY1-2) genes; their protein complexes return to the nucleus to suppress BMAL1 activity, while REV-ERBα and RORα fine-tune the timing and strength of BMAL1 expression to maintain circadian stability. Disturbances in the precision or strength of the molecular clock can impair its function, and epidemiological evidence links such circadian disruption to worsened outcomes in asthma, COPD, and pulmonary fibrosis (Maidstone et al., 2021[104]; Joshi and Sundar, 2023[75]). Recent studies show that clock-controlled genes (CCGs) in the lung are closely tied to inflammatory regulation in COPD. Analysis of bronchial epithelium revealed that reduced expression of core clock genes was inversely linked with heightened expression of pro-inflammatory mediators, while pathways supporting ciliated cell function were positively related to circadian activity. These findings highlight that disruption of circadian signaling contributes to abnormal lung inflammation, underscoring the importance of CCGs in maintaining pulmonary immune balance (Li et al., 2025[98]). 

The circadian clock in the lungs is essential for controlling airway tone, immune activity, and epithelial repair, thereby maintaining rhythmic homeostasis in pulmonary function. Disruption of this clock leads to heightened inflammatory signaling, weakened defense mechanisms, and impaired tissue repair, contributing to the progression of chronic respiratory diseases such as asthma and COPD, and increasing susceptibility to infections (Litinski et al., 2009[99]). In this context, BMAL1 in myeloid cells acts as a key negative regulator of allergic asthma by restraining lung inflammation and proinflammatory signaling, mitigating asthma severity through circadian modulation of immune cells (Zaslona et al., 2017[175]). Deletion of BMAL1 in mice alters the timing of respiratory cycles in a sex- and time-of-day-dependent manner, while overall ventilation remains largely unaffected (Jones and Arble, 2025[74]). This suggests that circadian disruption can subtly reshape respiratory rhythm generation without impairing baseline breathing, revealing sex- and time-specific vulnerabilities in respiratory control. Additionally, circadian rhythm disturbances exacerbate pulmonary fibrosis by upregulating DEC1 in alveolar type II epithelial cells, promoting p21-mediated cellular senescence and extracellular matrix deposition, which drives fibrotic progression (Chen et al., 2024[20]). Likewise, REV-ERBα is crucial in regulating pulmonary inflammation and epithelial-mesenchymal transition (EMT) induced by cigarette smoke. Loss of REV-ERBα worsens smoke-related circadian disruption and EMT, whereas its activation alleviates inflammation and abnormal EMT, indicating therapeutic potential for smoking-associated chronic lung diseases (Wang et al., 2021[166]). Collectively, these findings emphasize the role of circadian regulation in maintaining immune homeostasis and suggest that disruption of circadian timing can exacerbate inflammatory lung disorders such as asthma, COPD, and related conditions.

## Circadian Regulation of Lung Physiology

Circadian timing mechanisms play a fundamental role in coordinating lung function across the day-night cycle. By regulating airway tone, immune activity, secretion, and tissue repair, the internal clock ensures optimal respiratory performance and homeostasis. Disruption of these rhythms can exacerbate pulmonary dysfunction and increase disease susceptibility.

### Regulation of airway tone, immune responses, surfactant secretion, and repair

Circadian rhythms cause significant daily fluctuations in airway tone through complex interactions involving molecular clock genes, the autonomic nervous system, and immune-inflammatory processes. These variations have important physiological and clinical implications, especially in respiratory diseases such as asthma. Circadian rhythms modulate airway caliber in both healthy and asthmatic individuals, but in asthma, the enhanced bronchial hyper-responsiveness amplifies these oscillations, resulting in marked nocturnal bronchoconstriction and symptom manifestation (Barnes, 1985[10]). At the molecular level, airway cells harbor intrinsic circadian clocks composed of core clock genes such as CLOCK, BMAL1, PER, CRY, and the nuclear receptor REV-ERBα, which regulate time-of-day-dependent gene expression. REV-ERBα, in particular, plays a crucial role in gating airway smooth muscle contractility by repressing genes involved in muscle tone and inflammation. Animal studies have shown that deletion of REV-ERBα abolishes the normal daily rhythm of airway hyperresponsiveness, resulting in persistently elevated bronchoconstriction throughout the day (Durrington et al., 2020[35]). This finding indicates that the molecular clock shapes the temporal pattern of airway tone by controlling gene networks related to muscle contraction and inflammatory signaling. Circadian regulation extends to airway smooth muscle tone and muscarinic receptor expression, indicating that molecular clock mechanisms modulate bronchomotor control and airway responsiveness across the 24-hour cycle (Durrington et al., 2020[35]). Muscarinic receptor subtypes, particularly M2 and M3, play essential and distinct roles in regulating airway smooth muscle tone, making them critical pharmacological targets for effective management of obstructive airway diseases such as asthma and COPD (,and Jacoby, 1998[44]). This receptor rhythm partly explains the heightened airway constriction and tendency for asthma symptoms to worsen in the early morning hours. Timing therapeutic interventions to align with these receptor oscillations could improve drug efficacy, underlying the importance of circadian biology in respiratory pharmacotherapy.

Moreover, circadian clocks govern immune cells and inflammatory mediators in the airway, leading to rhythmic inflammation that indirectly affects airway tone. Circadian misalignment profoundly alters immune homeostasis, as the immune system itself exhibits intrinsic rhythmicity in cellular and humoral functions; disruption of these temporal patterns amplifies inflammatory responses and increases susceptibility to immune-mediated pathologies (Liu and Gillette, 1996[100]; Castanon-Cervantes et al., 2010[19]). This increases the level of cytokines such as IL-6 and IL-13, and immune cell infiltration rises rhythmically in the most common lung diseases, such as asthma, COPD, and fibroproliferative lung diseases (Holgate, 2008[64]; Castanon-Cervantes et al., 2010[19]; Provinciali et al., 2011[132]; Logan and Sarkar, 2012[101]). The mouse respiratory system contains SCN-driven peripheral clocks that regulate circadian expression of core clock and muscarinic receptor genes through parasympathetic (vagal) signaling. Consequently, mucus secretion from airway submucosal glands follows a circadian rhythm, reflecting coordinated neural and molecular control of respiratory physiology (Bando et al., 2007[8]). In mice, salivary IgA secretion follows a circadian rhythm controlled by the SCN through sympathetic norepinephrine signaling, acting via α- and β-adrenoceptors, and is independent of corticosterone, with rhythmicity lost after SCN lesions or clock gene mutations (Wada et al., 2017[161]). When the rhythmicity of salivary IgA secretion is lost due to SCN lesions or clock gene mutations, the time-of-day regulation of IgA release is disrupted, leading to a loss of coordinated mucosal immune defense in the salivary glands, which may impair the effectiveness of immune protection aligned with the circadian cycle. 

Circadian rhythms regulate lung repair by controlling the timing of key cellular activities involved in tissue regeneration (Naik et al., 2023[116]). Clock genes within lung cells orchestrate cycles of epithelial cell proliferation, immune cell function, and extracellular matrix remodeling, ensuring that repair processes occur at optimal times during the day (Hahn and Sundar, 2023[58]). This temporal regulation enhances the efficiency of healing and limits excessive inflammation. When these circadian controls are disrupted, repair is slowed, and tissue damage can worsen, emphasizing the critical role of the internal clock in coordinating lung recovery.

### Daily rhythmicity in lung function

Lung function exhibits a clear daily pattern, with peak expiratory flow (PEF) reaching its highest values during midday or afternoon and its lowest levels in the early morning, typically between 2 and 5 a.m. (Lebowitz, 1991[89]; Quackenboss et al., 1991[133]). This cycle is shaped by fluctuations in airway diameter and resistance, which become most constricted during the night and early morning, leading to lower PEF at those times. While these oscillations are usually minor in individuals without respiratory conditions, they are more pronounced in those with asthma or COPD, often mirroring periods of symptom worsening.

The underlying mechanisms for these rhythms involve increased parasympathetic nervous system activity at night, which narrows the airways, and coordination between central brain clocks and peripheral lung clocks. The central pacemaker in the brain communicates timing signals to lung tissues through the vagus nerve, while local lung cell clocks help regulate daily lung function at the tissue level (Bando et al., 2007[8]).

**Figure 4:**
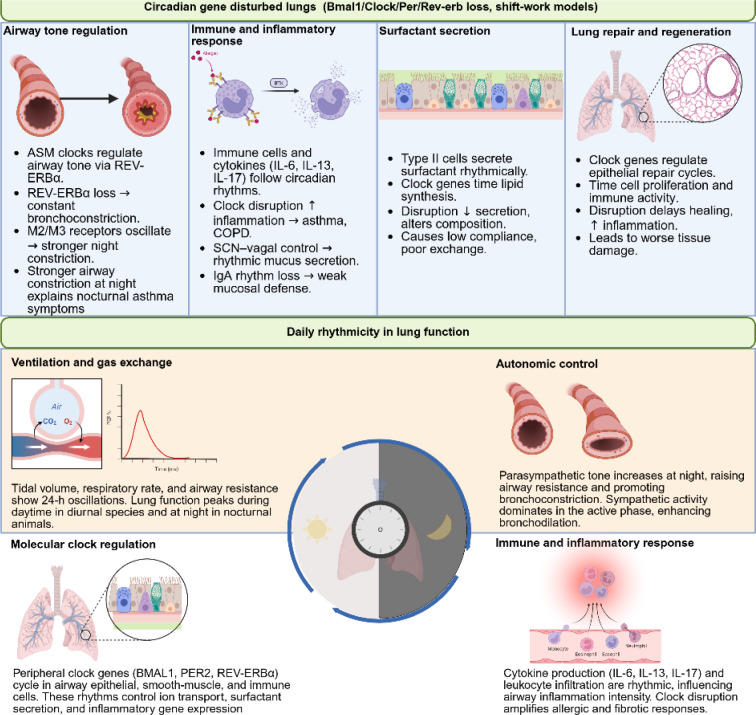
Circadian rhythms regulate lung physiology and how their disruption affects respiratory health. The top panel shows that loss of core clock genes (Bmal1, Clock, Per, Rev-erb) or shift-work-like conditions disturb local lung clocks, altering airway tone, immune balance, surfactant secretion, and tissue repair. Airway smooth-muscle clocks regulate bronchomotor tone through REV-ERBα and muscarinic receptors, with stronger nocturnal constriction leading to asthma symptoms. Immune and epithelial cells exhibit rhythmic cytokine and mucus secretion, while clock disruption heightens inflammation. Surfactant secretion by type II cells follows a circadian rhythm, and its disruption lowers compliance and gas exchange. Clock-controlled timing of epithelial repair governs regeneration; loss delays healing and promotes damage. The lower panel depicts daily rhythmicity in normal lungs ventilation and airway resistance oscillate over 24 h; parasympathetic tone rises at night, causing bronchoconstriction, while sympathetic tone dominates during active phases; peripheral clock genes (BMAL1, PER2, REV-ERBα) synchronize molecular and immune rhythms to optimize respiratory and inflammatory functions. Image was drawn using BioRender.com

This rhythmic behavior is clinically significant for tailoring diagnostic and therapeutic approaches, particularly in chronic respiratory diseases. Cosinor analysis shows that while the circadian peak of peak expiratory flow (PEF) remained in the early afternoon across all groups, both active and passive smoking significantly reduced the mean PEF (mesor) and increased its diurnal variability. Heavy smokers and COPD patients showed the lowest mesor and highest amplitude, while passive smokers had intermediate values. These findings suggest that increased PEF variability may serve as an early indicator of tobacco smoke-induced airway damage and COPD development (Casale and Pasqualetti, 1997[17]). Accounting for these time-of-day changes can help optimize management of lung health and disease. Circadian regulation of lung physiology and the effects of clock gene disruption on airway tone, immunity, surfactant secretion, and repair are summarized in Figure 4(Fig. 4).

## Disruption of Clock Genes and Circadian Rhythm in Lung Diseases

Disruption of circadian rhythms and core clock gene function has emerged as a critical contributor to the development and progression of lung diseases. Genetic alterations, environmental circadian misalignment, and lifestyle factors can desynchronize molecular clocks in lung tissues, leading to dysregulated inflammation, impaired repair, and altered immune responses. The following sections outline how circadian disruption influences major respiratory diseases through disease-specific mechanisms.

### Asthma

Asthma exhibits clear circadian patterns, with symptoms often worsening at night, a condition referred to as nocturnal asthma. This timing is driven by endogenous circadian rhythms, biological processes controlled by intrinsic molecular clocks composed of specific clock genes that regulate lung function and immune activity. Nocturnal asthma is defined by a drop in forced expiratory volume in one second (FEV1) of at least 15 % from bedtime to waking in individuals with clinical and physiological evidence of asthma, and in some cases, the reduction can exceed 20 % (Martin, 1999[106]). Nighttime exacerbation of asthma, including increased airway obstruction, wheezing, and coughing, aligns with circadian fluctuations in airway inflammation and bronchial hyperresponsiveness (Martin et al., 1990[108]; Kraft et al., 1996[84]; Van Keimpema et al., 1997[156]). During these hours, pro-inflammatory mediators such as interleukin-6 (IL-6) are elevated, intensifying airway inflammation (Reinhardt et al., 2019[136]). These oscillations are coordinated by core circadian proteins, including BMAL1 and CLOCK, which control gene expression in both structural lung cells and infiltrating immune cells. Altered expression or disruption of these clock genes impairs pulmonary immune homeostasis, amplifying inflammatory responses and worsening airway reactivity. For example, disruption of BMAL1, either genetically or through environmental circadian misalignment (e.g., jet lag), significantly enhances virus-induced airway inflammation and remodeling in mice, leading to increased mucus production, higher airway resistance, and reduced viral clearance (Ehlers et al., 2018[36]). 

These results indicate that the molecular circadian clock is crucial for preserving immune balance in the lungs, and its disruption can promote asthma-like conditions by altering antiviral defenses and airway epithelial function. In addition to the local lung clocks, the central circadian pacemaker in the suprachiasmatic nucleus coordinates peripheral lung clocks, ensuring the proper timing of physiological processes vital for respiratory health. Disruption of this coordination, whether due to environmental influences or genetic alterations, can impair asthma regulation and exacerbate symptoms.

### Chronic Obstructive Pulmonary Disease

COPD, primarily caused by cigarette smoking, affects around 10 % of adults over 40 and is marked by progressive small-airway obstruction leading to air trapping, hyperinflation, and exertional breathlessness, making it a major global cause of death and hospitalization (Barnes et al., 2015[11]). It is increasingly recognized as a condition influenced by disturbances in circadian biology (Yao et al., 2015[173]). The circadian system coordinates daily oscillations in lung physiology, immune defense, and inflammatory regulation. In individuals with COPD, these rhythms are often disrupted, leading to fluctuations in airway function, symptoms, and disease progression. Studies have shown that individuals with prominent morning symptoms have a greater likelihood of experiencing disease flare-ups and tend to rely more frequently on their rescue inhalers (Tsai et al., 2007[154]). One major contributing factor is cigarette smoking, which not only triggers oxidative stress and inflammation but also interferes with the molecular clock machinery that governs circadian rhythms (Hwang et al., 2014[69]).

Both experimental and clinical evidence indicate that smoking disrupts the expression of core circadian genes, including BMAL1, CLOCK, PER, and CRY, in airway epithelial cells and alveolar macrophages (Hwang et al., 2014[69]). This disturbance alters the timing of inflammatory mediator release, antioxidant defenses, and epithelial repair, contributing to the chronic inflammation and tissue remodeling seen in COPD. Additionally, circadian misalignment caused by irregular sleep-wake schedules, night-shift work, or exposure to environmental light can exacerbate these effects by desynchronizing lung peripheral clocks from the central pacemaker in the suprachiasmatic nucleus (Potter et al., 2016[130]; Boivin et al., 2022[13]). 

Together, smoking-induced oxidative stress and circadian disruption create a feedback loop that intensifies airway injury, weakens host defense against pathogens, and accelerates disease progression. Understanding how clock gene dysregulation contributes to COPD pathogenesis highlights the potential for chronotherapeutic strategies aimed at restoring circadian rhythm integrity to improve lung function and therapeutic outcomes in affected patients.

### Pulmonary fibrosis

Pulmonary fibrosis is a progressive lung disease marked by excessive extracellular matrix accumulation and scarring, which ultimately compromises gas exchange and respiratory function (Wang et al., 2024[163]). Recent studies indicate that dysfunction of the circadian clock contributes significantly to its development (Cunningham et al., 2020[25]; Chen et al., 2024[20]). Specifically, disruption of molecular clock components in fibroblasts and alveolar epithelial cells has been associated with impaired tissue repair and pathological fibrotic remodeling.

Fibroblasts, which are central to wound healing and matrix production, normally exhibit rhythmic activity regulated by clock genes such as BMAL1, CLOCK, and PER2 (Fawcett et al., 2022[41]). When these circadian pathways are dysregulated, fibroblasts display persistent activation and overproduction of collagen and other fibrotic proteins, promoting continuous scarring. Similarly, impaired circadian control in alveolar epithelial cells hampers timely regeneration and repair after lung injury, leading to chronic epithelial stress and activation of profibrotic signaling pathways, including TGF-β and Wnt (Zheng et al., 2025[180]).

Environmental factors such as disrupted sleep, altered light exposure, or systemic inflammation can further disturb circadian homeostasis, exacerbating fibroblast activation and tissue stiffness. Together, these findings indicate that the molecular clock serves as a key regulator of lung tissue integrity. Restoring circadian balance may therefore represent a promising therapeutic avenue to limit fibroblast-driven fibrosis and support proper epithelial repair in pulmonary fibrosis.

### Lung cancer

Lung cancer development and progression are increasingly associated with alterations in the circadian clock, which tightly regulates essential cellular processes such as the cell cycle, DNA repair, and metabolic homeostasis (Xiang et al., 2018[171]; Li et al., 2021[96]). The circadian system ensures that cell proliferation, energy metabolism, and genomic maintenance occur in a coordinated temporal manner (Panda, 2016[123]). Disruption of circadian regulation, whether due to genetic mutations, environmental misalignment, or lifestyle factors such as irregular light exposure and night-shift work, can create conditions that favor tumor development (Dobrovinskaya et al., 2024[30]). Core clock genes, including BMAL1, CLOCK, PER, and CRY, interact with key oncogenic and tumor suppressor pathways, such as p53, MYC, and KRAS, to regulate cell proliferation and apoptosis (Jiang et al., 2016[73]; Kontomanolis et al., 2020[83]; Burchett et al., 2021[14]). When these clock components are dysregulated, DNA damage repair is compromised, cell division becomes uncontrolled, and metabolic signaling is altered, collectively promoting malignant transformation and cancer progression. In the lungs, disrupted circadian timing has been associated with elevated oxidative stress, genomic instability, and accelerated tumor growth. (Zhao et al., 2025[179]).

Moreover, circadian misalignment can affect the tumor microenvironment by altering immune surveillance, angiogenesis, and the timing of drug metabolism, potentially influencing treatment outcomes (Li et al., 2024[93]). These findings highlight the circadian clock as a key modulator of cancer biology and suggest that aligning therapeutic interventions with circadian rhythms, known as chronotherapy, could optimize efficacy and reduce toxicity in lung cancer management.

### Acute Lung Injury/ARDS

Acute Lung Injury (ALI) and its severe form, Acute Respiratory Distress Syndrome (ARDS), are characterized by excessive inflammation, disruption of the alveolar-capillary barrier, pulmonary edema, and compromised gas exchange (Xie et al., 2025[172]). Emerging evidence suggests that circadian rhythms critically influence the timing and intensity of these inflammatory and reparative processes (Baxter and Ray, 2020[12]). The concept of circadian gating proposes that immune activation and tissue repair fluctuate throughout the 24-hour cycle, affecting both the severity of the disease and the efficiency of recovery (Labrecque and Cermakian, 2015[87]).

At the cellular level, Circadian clock genes such as BMAL1, CLOCK, PERs, CRYs, REV-ERBs, and RORs govern both innate and adaptive immune behaviors by controlling the rhythmic transcription of chemokines, cytokines, and immune receptors, with each gene exerting a specific influence on inflammatory states and leukocyte migration patterns. For example, BMAL1 and CLOCK heterodimers rhythmically repress Ccl2, Ccl8, S100a8, and Tlr9, promoting time-of-day-dependent immune cell trafficking, while REV-ERBα downregulates Nlrp3 and Il6 to dampen inflammasome activity, and RORα modulates IkBa expression to inhibit NFκB-driven inflammatory signaling, altogether ensuring temporal gating of immune function and disease susceptibility (Hergenhan et al., 2020[63]). PER2 integrates light-regulated circadian signaling and metabolic adaptation in alveolar epithelial cells by stabilizing HIF1, promoting efficient carbohydrate and fatty acid metabolism, and enhancing cellular resilience to mechanical stretch during ventilator-induced lung injury; intense light exposure further increases pulmonary PER2 levels, positioning alveolar HIF1 as a potential therapeutic target for PER2-driven treatment strategies in acute lung injury (Sundar et al., 2015[151]).

Clinically, these findings suggest that circadian timing could be leveraged to optimize treatment strategies for ALI and ARDS. Chronotherapy, administering anti-inflammatory drugs, corticosteroids, or mechanical ventilation in alignment with circadian phases, may enhance efficacy and reduce tissue damage. Overall, the circadian clock emerges as a key regulator of pulmonary immune homeostasis, and its disruption contributes significantly to the onset, persistence, and outcome of acute lung injury.

### Sleep-disordered breathing

**Figure 5:**
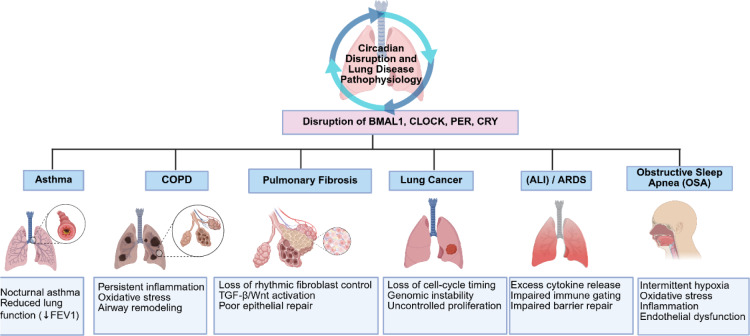
Circadian dysregulation impairs immune regulation, epithelial repair, and metabolic balance, leading to disease-specific effects: nocturnal inflammation in asthma, persistent oxidative stress in COPD, fibrotic remodeling in pulmonary fibrosis, uncontrolled proliferation in lung cancer, excessive inflammation in ALI/ARDS, and systemic dysfunction in OSA. Overall, circadian misalignment is a key contributor to lung disease pathophysiology. Image was drawn using BioRender.com.

Obstructive sleep apnea (OSA) is characterized by repeated upper airway collapse during sleep, causing reduced or halted airflow, oxygen desaturation, and fragmented, non-restorative sleep, often accompanied by loud snoring, observed breathing pauses, and daytime fatigue (Sankri-Tarbichi, 2012[138]; Carneiro-Barrera et al., 2019[16]; Esteller et al., 2019[37]; Mehrtash et al., 2019[111]). OSA is increasingly understood as a disorder that disrupts the body's intrinsic circadian clock, with broad systemic effects (Malicki et al., 2022[105]). Patients with OSA frequently experience circadian misalignment due to intermittent hypoxia, fragmented sleep, and irregular respiratory patterns (Patel, 2019[126]; Rundo, 2019[137]; Gabryelska et al., 2022[46]). This disruption compromises the coordination of molecular clocks that regulate physiological processes across nearly all organs, thereby worsening metabolic, cardiovascular, and neurodegenerative conditions (Monahan and Redline, 2011[115]; Gonzaga et al., 2015[54]; Andrade et al., 2018[5]; Li et al., 2018[97]; Dredla and Castillo, 2019[33]; Song et al., 2019[149]; Gabryelska et al., 2021[45]). Recent studies suggest that circadian clock disruption may not only result from OSA but also contribute to the development of its systemic complications, highlighting the circadian system as a potential target for therapeutic strategies to reduce disease burden in OSA patients. Disruption of core circadian clock genes drives distinct pathological pathways across major lung diseases (Figure 5(Fig. 5)).

## Mechanistic Insights: Linking Clock Genes to Pathophysiology

Disruption of circadian timing alters fundamental molecular pathways that maintain lung homeostasis. Core clock genes directly influence inflammatory signaling, metabolic regulation, and cellular stress responses, linking temporal control to disease mechanisms. This section focuses on how clock gene dysfunction drives pathological processes in the lung through defined molecular interactions.

### Role of BMAL1, CLOCK, PER, CRY, REV-ERBα/β, RORα in lung disease models

In lung tissues, the BMAL1-CLOCK heterodimer drives rhythmic expression of clock-controlled genes involved in airway tone, epithelial barrier integrity, immune cell trafficking, and oxidative stress responses. Reduced BMAL1 and CLOCK activity appears to favor stress‑induced senescence in bronchial epithelial cells *via* MAPK signaling, which may contribute to accelerated lung aging and COPD progression (Li et al., 2022[95]). Similarly, circadian disruption through Bmal1 deficiency or environmental jet lag intensifies virus-induced bronchiolitis and asthma-like airway remodeling in mice, highlighting clock gene regulation of antiviral defenses in the lung (Ehlers et al., 2018[36]). Likewise, Respiratory syncytial virus (RSV) infection disrupts clock gene rhythms and amplifies inflammation *via* the p38 MAPK pathway, with BMAL1 disruption emerging as a key driver of exacerbated lung pathology in both mouse models and human bronchial cells (Wang et al., 2025[165]). Recent work has shown that the circadian repressor Rev-Erbα curbs myofibroblast differentiation along with collagen production in both fibroblast cultures and fibrotic lung tissue from patients with pulmonary fibrosis (Raza et al., 2022[135]). Lung tissues from emphysema-afflicted mice exhibited diminished Rev-Erbα gene and protein levels (Hwang et al., 2014[69]). Notably, Rev-Erb−/− mice showed elevated markers of pulmonary myofibroblast activation, including collagen-1 and α-SMA (Cunningham et al., 2020[25]). These insights collectively emphasize the protective functions of core clock components BMAL1, CLOCK, REV-ERBα/β, and others like PER, CRY, and RORα across diverse lung disease models, from senescence and viral infections to fibrosis and emphysema, suggesting their therapeutic modulation as a frontier in respiratory chronobiology.

### Interaction with inflammatory pathways

Bmal1 regulates time-dependent Toll-Like Receptor 4 (TLR4) -mediated inflammatory responses in macrophages by controlling RevErb-dependent enhancer activity and epigenetic states, thereby shaping circadian regulation of inflammation (Sato et al., 1999[140]). In addition to the direct control of inflammatory gene expression by clock components, circadian regulation of inflammation is also mediated through the glucocorticoid receptor (GR). Rhythmic secretion of endogenous glucocorticoids activates intracellular GR signaling, which suppresses inflammatory cytokine expression via glucocorticoid response elements and through transrepressive interactions with key transcription factors such as NF-κB and Activator protein-1 AP-1 (Fagiani et al., 2022[40]). Consistent with this mechanism, a cell-intrinsic circadian clock in airway epithelial club cells regulates pulmonary immune homeostasis by coordinating glucocorticoid receptor-dependent control of CXCL5 expression, thereby driving time-of-day-specific neutrophil recruitment; disruption of Bmal1 exaggerates inflammatory responses and compromises antibacterial host defense (Gibbs et al., 2014[51]). Beyond airway epithelial cells, emerging evidence indicates that stromal fibroblasts also engage circadian mechanisms to modulate pulmonary inflammation. In lung fibroblasts, BMAL1 acts as a brake on inflammatory signaling by limiting NF-κB activity, thereby constraining chemokine output and neutrophil recruitment in response to inflammatory stimuli. Disruption of this fibroblast-intrinsic clock enhances CXCL5 production and amplifies neutrophil migration, highlighting a complementary, cell-type-specific layer of circadian control over lung immune responses (Cox et al., 2023[22]). This interplay between circadian regulation and immune activation creates a self-reinforcing loop, where clock gene dysfunction sustains inflammation and contributes to the progression of inflammatory and degenerative lung diseases, highlighting the circadian-immune interface as a promising target for therapeutic intervention. 

### Crosstalk with oxidative stress and metabolic regulation

Clock genes are essential not only for sustaining circadian rhythms but also for regulating cellular metabolism and redox balance (Mazzoccoli et al., 2012[109]). Disruption of these clocks can impair mitochondrial function, leading to elevated reactive oxygen species and oxidative stress (Sardon Puig et al., 2018[139]; Mezhnina et al., 2022[113]). Key metabolic regulators, including AMP-activated protein kinase (AMPK), sirtuin 1 (SIRT1), and peroxisome proliferator-activated receptor gamma coactivator-1α (PGC-1α), are closely linked to circadian cycles, and their dysregulation contributes to disrupted energy homeostasis, impaired autophagy, and increased susceptibility to metabolic and inflammatory diseases (Nogueiras et al., 2012[122]; Sato and Sato, 2023[141]). Oxidative stress can, in turn, influence the expression and activity of core clock genes, establishing a bidirectional relationship between circadian regulation and cellular metabolism (Patel et al., 2014[125]). This interaction is particularly important in metabolically active or inflammation-prone tissues such as the lung, liver, and heart, where circadian disruption amplifies oxidative damage, promotes inflammatory signaling, and heightens disease risk. Overall, these findings underscore the role of circadian clocks as central integrators of metabolic and redox cues, linking temporal regulation to cellular homeostasis and disease susceptibility.

## Therapeutic Targeting of Circadian Clocks in Lung Diseases

Growing understanding of circadian biology has revealed the internal clock as a modifiable factor in lung disease treatment. Therapeutic strategies that restore or exploit circadian timing can improve drug efficacy, reduce toxicity, and enhance disease control. This section discusses chronotherapy, pharmacological clock modulation, lifestyle-based interventions, and their integration into precision medicine approaches for respiratory disorders.

### Chronotherapy

Chronotherapy is a treatment strategy that aligns drug administration with the body's intrinsic circadian rhythms to enhance therapeutic efficacy and minimize adverse effects (Kaur et al., 2013[78]). In respiratory medicine, this approach is especially important because lung physiology, airway responsiveness, inflammatory processes, and immune cell movement all follow circadian patterns. Timing pharmacological interventions to correspond with daily fluctuations in lung function and disease activity provides a targeted framework for improving outcomes in chronic pulmonary disorders while reducing systemic side effects.

Asthma symptoms often worsen in the early morning period. They are accompanied by a simultaneous reduction in lung function, underscoring the clinical significance of administering therapies in a time-dependent manner. Symptoms are most frequently observed between midnight and early morning, with maximal severity occurring near 4 a.m. (Martin and Banks-Schlegel, 1998[107]). This concept has promoted the adoption of chrono pharmacological strategies in asthma treatment and the development of drugs tailored to circadian changes in airway physiology, where most regimens rely on once-daily nighttime dosing to suppress nocturnal airway inflammation and prevent early-morning airflow limitation, and where, in addition to bronchodilators, once-daily inhaled glucocorticosteroids have been introduced to enhance nighttime symptom control while also improving patient adherence, with numerous clinical investigations supporting the efficacy of chronotherapy, particularly in patients with nocturnal asthma (Burioka et al., 2010[15]). In COPD, addressing diurnal airflow limitation with sustained 24-hour bronchodilation particularly tiotropium combined with formoterol produces the greatest improvements in daytime and nocturnal FEV₁ while reducing rescue medication use compared with monotherapy (Van Noord et al., 2005[158], Moita et al., 2008[114]). Circadian features and chronotherapeutic approaches across major pulmonary diseases and lung-associated cancers are summarized in Table 1(Tab. 1) (References in Table 1: Cox et al., 1984[21]; Extra et al., 1990[39]; Gunn et al., 1995[57]; Huang et al., 2025[66]; Moita et al., 2008[114]; Pekovic-Vaughan et al., 2014[127]; Shang and Li, 2023[145]; Tamimi et al., 2020[152]; Truong et al., 2016[153]; van Noord et al., 2005[158]; Vandeleur et al., 2017[159]; Veale et al., 1994[160]).

### Pharmacological modulation of clock genes

Targeting components of the circadian clock has emerged as a promising therapeutic strategy for modulating lung inflammation and fibrosis. REV-ERB and ROR nuclear receptors coordinate circadian, metabolic, and immune functions, and the identification of their endogenous ligands has highlighted them as druggable targets with wide-ranging therapeutic potential (Kojetin and Burris, 2014[81]). Agonists of Rev-erbα have been shown to inhibit TGFβ1-induced fibroblast-to-myofibroblast differentiation, extracellular matrix production, and pro-inflammatory signaling, underscoring their potential in treating fibrotic lung diseases such as IPF (Prasad et al., 2023[131]). Conversely, reduced REV-ERBα expression can promote lung cancer progression by enhancing NF-κB signaling, increasing cell proliferation and invasion, pointing to REV-ERBα as a therapeutic target (Zhang et al., 2022[177]). Age-related circadian disruptions, characterized by altered Rev-erbα expression and changes in the apelin/apelin receptor pathway, compromise pulmonary host defenses and raise susceptibility to bacterial respiratory infections. Pharmacological activation of Rev-erbα in aged mice restores immune function and enhances resistance to infection (Silva Angulo et al., 2025[148]). Additionally, REV-ERBs act as negative regulators of liver fibrosis by suppressing NLRP3 inflammasome activity, with pharmacological activation reducing fibrogenesis (Wang et al., 2025[164]).

Conversely, Synthetic ROR agonists and inverse agonists, particularly targeting RORγt to suppress IL-17 production, show promise as therapeutic strategies for autoimmune, inflammatory, metabolic, and other ROR-related diseases (Jetten et al., 2021[72]). RoR2 enhances Wnt3a-driven canonical signaling in lung carcinoma cells via Fzd2-dependent interactions, revealing its novel role in modulating canonical Wnt pathways (Li, Chen et al., 2008[92]). REV-ERBα acts as a negative regulator of Th17 cell development by competing with RORγt, suppressing pro-inflammatory cytokine expression, and its pharmacological modulation reduces autoimmune disease severity (Amir et al., 2018[4]). In summary, ROR-directed modulators influence immune homeostasis by shaping Th17 cell responses, macrophage phenotypes, and epithelial immune signaling, thereby contributing to disease susceptibility and progression in asthma, COPD, and pulmonary fibrosis. 

Furthermore, circadian timing is governed by light-induced **Per1/Per2** transcription and CK1δ/ε-dependent post-translational regulation of PER stability, nuclear translocation, and PER-CRY interactions; however, selective inhibition of CK1ϵ has minimal effects, indicating that CK1δ is the dominant regulator of the mammalian circadian clock, while CK1ϵ inhibitors remain valuable tools for dissecting kinase-specific signaling roles (Gallego and Virshup, 2007[48]; Walton et al., 2009[162]). Building on this, another study has shown that pharmacological inhibition of CK1δ/ε, such as with PF-670462, can reset circadian rhythms in a timing-dependent manner, with early-day dosing producing stable phase delays, emphasizing that both drug timing and environmental light cues must be carefully considered for effective chronopharmacological modulation of circadian rhythms (Kim et al., 2013[79]).

### Lifestyle interventions

Environmental and behavioral cues play a critical role in regulating circadian rhythms, offering potential therapeutic avenues for lung diseases. Photobiomodulation reduces lung inflammation, Th2 cytokines (IL-4, IL-5, IL-13), enhances muscle metabolism, and promotes angiogenesis, sleep, and airway remodeling, making it a promising adjunct therapy for COPD (Lu et al., 2023[103]; Jung and Kim, 2024[76]; Cruz et al., 2025[24]; Gaggi et al., 2025[47]; Mehdizadeh et al., 2025[110]). The central SCN clock coordinates peripheral circadian oscillators to regulate physiology and behavior, and disruption of these rhythms increases the risk of metabolic, inflammatory, and cognitive disorders (Crespo et al., 2025[23]). Maintaining good sleep habits, such as consistent sleep schedules and minimizing nighttime light, supports circadian stability, enhances sleep quality, and may benefit overall lung function (Baranwal et al., 2023[9]). Together, these lifestyle strategies provide non-drug approaches to support circadian regulation in lung health.

### Potential for precision medicine

Advances in chronobiology have highlighted the promise of circadian biomarkers in guiding personalized therapy for lung diseases. Variations in the expression patterns of clock genes and downstream circadian-regulated pathways can influence both disease progression and drug responsiveness (Almon et al., 2008[3]). By profiling these temporal signatures in individual patients, clinicians may identify subgroups more likely to benefit from specific treatments, including time-optimized drug administration. This approach enables tailoring therapeutic strategies to each patient's intrinsic circadian rhythm, potentially improving efficacy while minimizing adverse effects. Incorporating circadian biomarkers into clinical practice could therefore represent a significant step toward precision medicine in respiratory disorders, allowing interventions to align with the body's biological timing for maximal therapeutic impact.

## Challenges and Future Perspectives

Chronotherapy and circadian medicine offer promising strategies for improving treatment outcomes in lung diseases, but several challenges must be addressed for successful clinical translation. A major issue is the translation from animal models to human disease. While rodent studies have illuminated circadian regulation of gene expression, immune responses, and drug metabolism, differences in physiology, sleep-wake patterns, and disease complexity limit direct applicability. Timing that is effective in nocturnal animals may not correspond to optimal therapeutic windows in humans. Moreover, human lung diseases are heterogeneous, involving genetic, environmental, and comorbid factors that are difficult to replicate in preclinical models. Bridging this gap requires validation in human tissues and well-designed clinical trials incorporating time-of-day dosing.

Individual variability in circadian biology adds another layer of complexity. Genetic factors, age, lifestyle, and sleep habits cause differences in circadian rhythms between patients, affecting both disease progression and drug responsiveness. Developing reliable circadian biomarkers can help stratify patients and personalize treatment schedules, ensuring therapies align with each individual's biological timing. The integration of circadian medicine into clinical practice presents logistical and educational challenges. Time-specific dosing may conflict with conventional hospital routines or patient adherence, necessitating user-friendly tools for monitoring circadian markers and educating healthcare providers on chronotherapy principles.

Finally, there is significant potential for drug discovery targeting clock pathways. Clock proteins such as BMAL1, CLOCK, PER, CRY, and REV-ERBs regulate processes relevant to lung health, including inflammation and tissue repair. Pharmacological modulation of these pathways offers new avenues for therapy but requires careful assessment to avoid systemic side effects. High-throughput screening and computational modeling will be key to identifying safe and effective clock-targeting compounds. Addressing these challenges, translating preclinical findings, accounting for individual circadian differences, integrating timing-based interventions into clinical care, and exploring clock-targeted therapies will be crucial for realizing the full potential of circadian medicine in lung disease.

## Conclusion

Clock genes are key regulators of lung function and respiratory health, modulating immune responses, tissue repair, and cellular metabolism. Disruption of circadian rhythms has been linked to the onset and progression of lung conditions such as asthma, chronic obstructive pulmonary disease, and interstitial lung disorders. Such disturbances can impact inflammatory signaling, airway dynamics, and the efficacy of medications, highlighting the importance of maintaining circadian alignment in pulmonary physiology. Targeting circadian pathways presents a promising therapeutic avenue to enhance treatment outcomes, optimize drug timing, and minimize side effects. Integrating circadian biology into clinical practice may enable personalized, time-based interventions, offering a novel strategy for improving patient care and managing respiratory diseases.

## Notes

Kamal Dua and Keshav Raj Paudel (Dr. PhD, Co-Lead Respiratory and Pharmaceutical Research Team, NICM Health Research Institute and School of Science, Western Sydney University NSW 2145, Australia; E-mail: k.paudel@westernsydney.edu.au) contributed equally as corresponding author.

## Declaration

### Funding 

No funding was received for this study.

### Availability of data and materials 

Not applicable. 

### Ethics approval and consent to participate. 

Not applicable. 

### Consent for publication

All authors have approved to publish this manuscript. 

### Competing interests 

All authors declare no conflict of interest.

### Artificial Intelligence (AI) - assisted technology

AI-assisted technology (ChatGPT) was used exclusively for language refinement, with no role in the development of the manuscript's scientific or original content.

## Figures and Tables

**Table 1:**
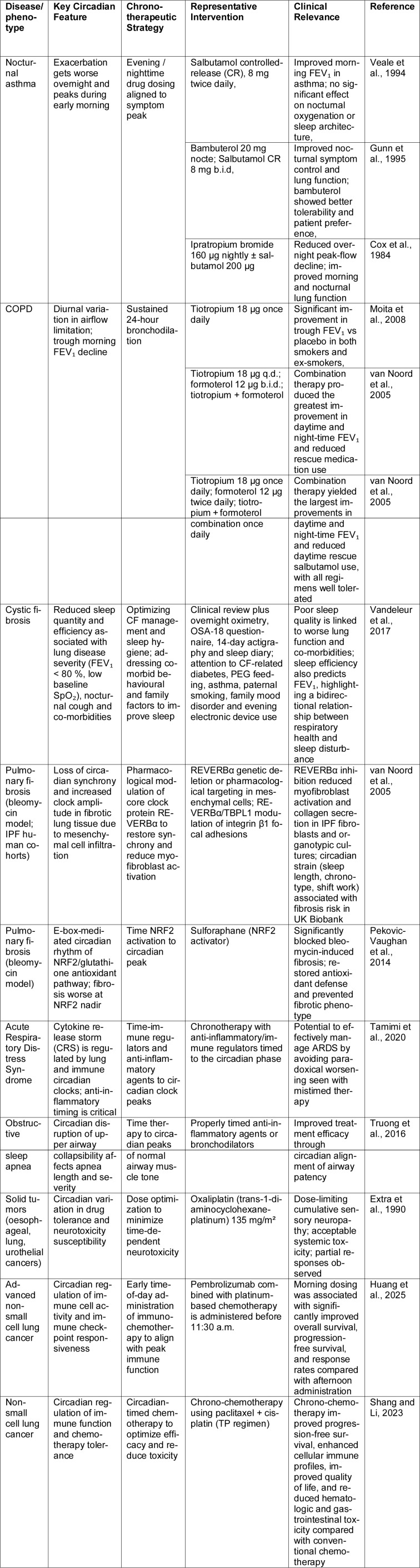
Circadian features and chronotherapeutic strategies across pulmonary diseases and lung-associated malignancies, highlighting time-of-day-dependent pathophysiology, representative interventions, and their clinical relevance.

**Figure 1:**
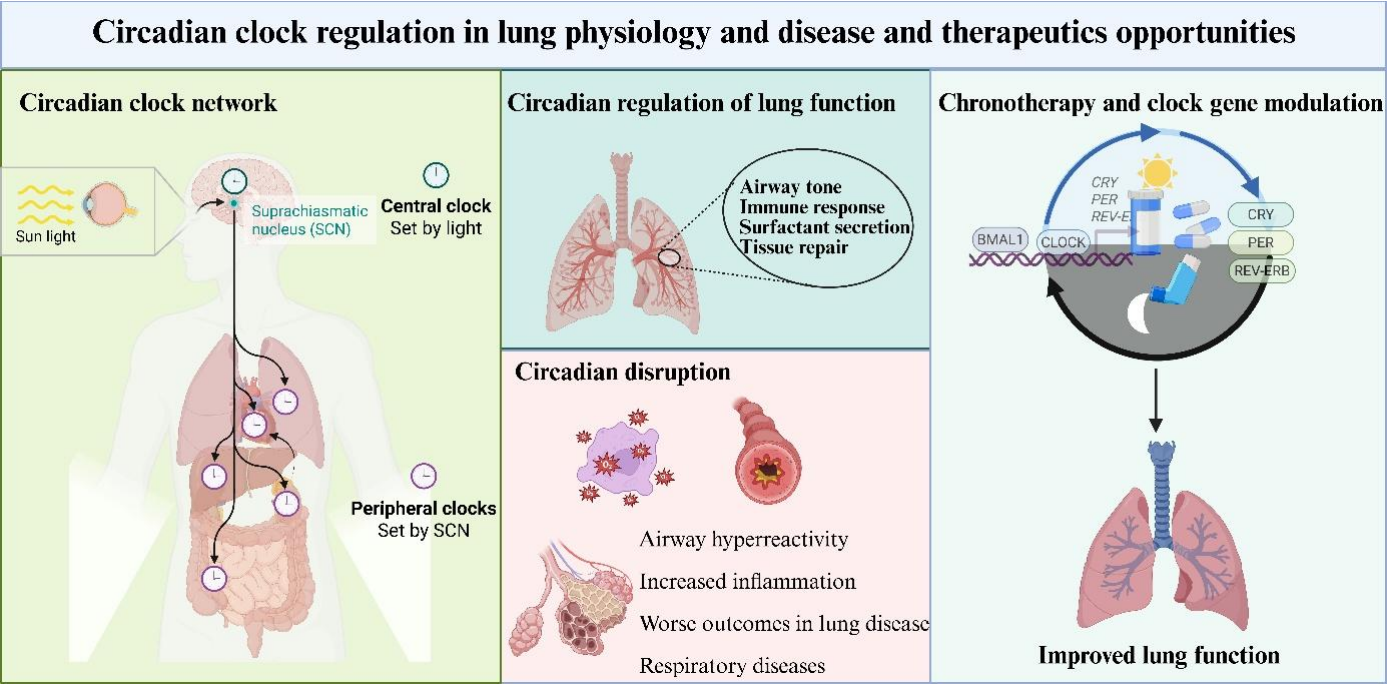
Graphical abstract
